# Magnetoelectric Composites: Applications, Coupling Mechanisms, and Future Directions

**DOI:** 10.3390/nano10102072

**Published:** 2020-10-20

**Authors:** Dhiren K. Pradhan, Shalini Kumari, Philip D. Rack

**Affiliations:** 1 Department of Materials Science & Engineering, University of Tennessee, Knoxville, TN 37996, USA; 2 Center for Nanophase Materials Sciences, Oak Ridge National Laboratory, Oak Ridge, TN 37831, USA; 3 Department of Materials Science & Engineering, The Pennsylvania State University, University Park, PA 16802, USA; szk1009@psu.edu

**Keywords:** ferroelectricity, magnetism, magnetoelectric coupling, strain, exchange bias

## Abstract

Multiferroic (MF)-magnetoelectric (ME) composites, which integrate magnetic and ferroelectric materials, exhibit a higher operational temperature (above room temperature) and superior (several orders of magnitude) ME coupling when compared to single-phase multiferroic materials. Room temperature control and the switching of magnetic properties via an electric field and electrical properties by a magnetic field has motivated research towards the goal of realizing ultralow power and multifunctional nano (micro) electronic devices. Here, some of the leading applications for magnetoelectric composites are reviewed, and the mechanisms and nature of ME coupling in artificial composite systems are discussed. Ways to enhance the ME coupling and other physical properties are also demonstrated. Finally, emphasis is given to the important open questions and future directions in this field, where new breakthroughs could have a significant impact in transforming scientific discoveries to practical device applications, which can be well-controlled both magnetically and electrically.

## 1. Introduction

**Figure 1:**
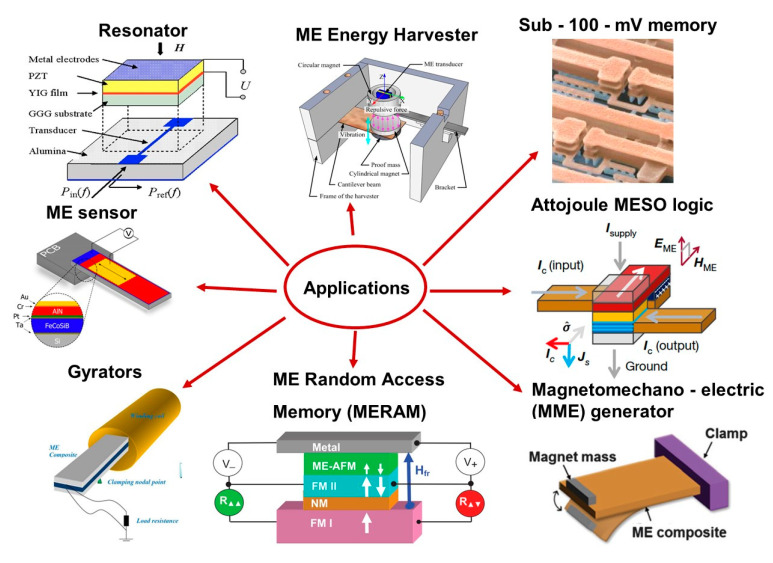
Various applications of magnetoelectric (ME) composites. Figures reproduced with permission from [9,39,40,41,43,44,45].

In recent years, with the trends towards miniaturizing devices to enhance the speed and reduce power and cost, there is a great need to combine the electrical and magnetic properties in a single-phase material [1,2,3,4,5]. Multiferroic (MF) materials are special classes of materials, which display two or more ferroic orderings, such as ferroelectricity (FE), ferro(antiferro/ferri) magnetism, ferrotoroidicity, and ferroelasticity in a single-phase [2,6,7,8,9]. The presence of several ferroic parameters in a single-phase material leads to novel physical phenomena [1,2]. Magnetoelectric (ME) coupling originates due to the coupling between FE and FM ordering parameters, where polarization (*P*) can be switched and/or tuned by a magnetic field (*H*) and magnetization (*M*) can be manipulated and/or switched via an electric field (*E*) [2,6,9,10]. ME-MF materials are rich in fundamental physics and they have great potential for applications in novel devices, such as ultra-low power and highly dense logic-memory, radio- and high-frequency, micro(nano) electronic, sensors, energy harvesting, actuators, spintronics, miniature antennas, terahertz emitters, electric-field controlled FM resonance, and other multifunctional devices [2,4,6,7,9,11,12,13,14,15,16,17]. The coexistence of electric polarization and magnetization in a single-phase makes them suitable for multistate memory devices, as *P* and *M* are used to encode binary information in FRAM (FE random-access memories) and MRAM (magnetic random-access memories), respectively [5,13,16,18,19,20]. Magnetoelectric RAM (MERAM) combines the characteristics of both FRAM and MRAM along with additional functionalities [16]. ME materials that are utilized in ME spin orbit (MESO) logic devices show lower switching voltage, higher switching energy, and superior logic density than the existing complementary metal–oxide–semiconductor (CMOS) based devices [4,9]. Quite few ME materials exist in nature because of incompatibility between ferroelectric and magnetic orderings in oxides [5,21]. Some well-known single-phase MF materials are: BiMnO_3_, YMnO_3_, BiFeO_3,_ TbMnO_3_, Pb(Fe_0.5_Nb_0.5_)O_3_, Pb(Fe_0.67_W_0.33_)O_3_, Fe_3_O_4_, GaFeO_3_, Pb(Zr_0.20_Ti_0.80_)_0.70_Pd_0.30_O_3-δ_, TbMnO_3_, PbTi_0.90_Pd_0.10_O_3_, YbMnO_3_, etc. [1,2,6,22]. The majority of the single-phase MF materials have a magnetic transition temperature (*T_C_*) below RT. Because of the large difference between the FE and magnetic *T_C_*, the ME coupling is observed to be very small [17,22,23]. For practical applications, MF materials should exhibit large polarization and magnetization, along with strong ME coupling at room temperature (RT) [3,4,5,9,19]. The single-phase ME materials discovered to date are not suitable for the envisioned devices due to their low ME coupling coefficient, and lower operational temperature [3,9,15,16,23]. To overcome these drawbacks, doping in A-site, B-site and/or both the sites in single-phase materials with suitable dopants have been attempted [3,15,24,25,26,27,28,29,30,31]. The ME coupling and other physical parameters have been enhanced, but are not suitable for practical device applications [5,9,23,32]. Artificial ME composite structures have been investigated in order to observe strong ME coupling and transition temperatures (ferroelectric and magnetic) above RT [12,32,33,34,35]. ME composite structures consist of ferroelectric materials having large polarization and high piezoelectric and/or electro-strictive coefficients, higher *T_C_* (well above RT) and magnetic materials exhibiting large magnetization, *T_C_* well above RT, high resistivity, and large piezomagnetic and/or magnetostrictive coefficients can exhibit large ME coupling at RT [9,22,33,36,37]. ME composites exhibit high polarization and magnetization, large ME coupling, low loss tangent, and lower switching voltage at RT, along with critical temperatures above RT, the flexibility in size, materials design, and manufacturability. The magnetic and ferroelectric states can be manipulated and switched by *E* and *H,* respectively, and require relatively lower energy. ME composites also exhibit tunability behavior, which originates due to the shifting of the impedance, resonant frequency, and output voltage with the bias [1,2]. The ME coefficient is a few orders of magnitude higher at resonant frequencies than the sub-resonant frequencies. These outstanding characteristics make them suitable for different applications, such as ultralow power logic–memory devices, high energy density capacitors, magnetic field sensors, gyrators, spin-filters, resonators, EMI shielding, phase shifters, inductors, biomedical applications, magnetoelectric antenna, terahertz emitters, transducers, spintronics, and other multifunctional devices [4,9,11,13,16,32,37,38,39,40]. MESO logic devices utilize the Rashba–Edelstein and ME (E-field control of magnetization) effects and exhibit lower switching voltage and higher logic densities [4,9]. Magneto-mechano-electric (MME) generators contain a piezoelectric material on a magnetostrictive metal plate in the form of a cantilever structure. When an AC magnetic field is applied to the MME generator, the magnetostrictive layer of the ME composite elongates or contracts (magneto-mechano coupling), which induces strain in the piezoelectric materials and results in an output voltage across the electrical load via direct piezoelectric effects [41]. ME random access memory (MERAM) cells consist of a ME–antiferromagnet exchange coupled with a switchable ferromagnetic material, a tunneling barrier (nonmagnetic), and a hard ferromagnetic material. MERAM exhibit multistate memory elements and consume ultralow power [40,42]. ME composites are suitable candidates to be utilized in gyrators, as they possess higher ME coupling and higher magneto-mechanical conversion capability [43]. The ME resonators utilize the E- field tunability of the ferromagnetic resonance (FMR) absorption [39]. ME sensors having FeCoSiB as a magnetic layer and AlN as a FE layer can reveal low detection limits on the order of ~pT/Hz^1/2^ in mechanical resonance [44]. ME energy harvesters convert vibration energy to electrical energy and utilize electromagnetic and ME energy harvesting principles at low vibration frequencies and deliver energy with maximum efficiencies [45]. Figure 1 presents some important applications of the ME composites. These ME devices having reduced dimensions will enhance the speed and reduce their associated power and cost.

**Figure 2:**
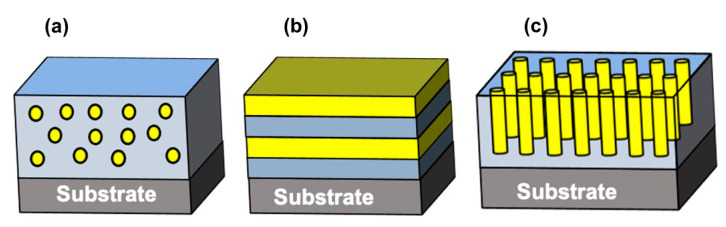
Schematic of (**a**) 0-3, (**b**) 2-2, and (**c**) 1-3 composite nanostructures.

In order to enhance the ME coupling and other required physical properties in composites, appropriate composite architectures have been studied [32,46]. There are several composite architectures that can be designed based on different types of connectivity of ferroelectric and magnetic materials. The possible composite architecture depending on the connectivity are: 0-0, 0-1, 1-1, 2-1, 3-1, 3-2, 3-3, 0-2, 1-2, 2-2, and some hybrid architectures, where the number implies the dimension of the magnetic and ferroelectric components, respectively [32,46]. The most commonly used ME architectures with successfully proven enhanced physical properties are: 0-3 particulate composites (where the magnetic nanoparticles (0-dimensional) are dispersed in ferroelectric matrices (three-dimensional)), 1-3 rod composites (where one-dimensional rods are grown in three-dimensional ferroelectric matrix), and 2-2 layered composites (where two-dimensional magnetic thick/thin layers are grown on two-dimensional ferroelectric thin films, and the periodicity might more than one) [17,32,46,47,48,49]. Figure 2 shows the 0-3, 1-3, and 2-2 composite nanostructures.

In order to observe large ME coupling, various types of composite systems have been synthesized, such as (a) FE-FM (antiferro/ferrimagnetic) composites, (b) MF-MF composites, (c) MF-FM (antiferro/ferrimagnetic) composites, and (d) FE-MF composites [15,17]. Most of the composite architectures have been designed in different connectivity utilizing Pb(Fe_0.5_Nb_0.5_)O_3_, BaTiO_3_, Ba_1−x_Sr_x_TiO_3_, BiFeO_3_, Pb(Zr_1−x_Ti_x_)O_3_, and PbTiO_3_ as FE candidates and La_1−x_Sr_x_MnO_3_, Fe_3_O_4_, CoFe_2_O_4,_ NiFe_2_O_4_, Ni_1−x_Zn_x_Fe_2_O_4_, and Co_1−x_Zn_x_Fe_2_O_4_ as magnetic component [15,34,36,50,51,52,53,54,55,56,57,58,59,60,61,62].

## 2. ME Effect

The discovery of Maxwell’s equations in 1865 suggested the intimate relationship between electricity and magnetism, which were initially believed to be two independent phenomena [10,19]. After 30 years, Pierre Curie in 1894 proposed the linear relationship between the electrical and magnetic properties i.e., some materials exist in nature, which can be magnetized by an electric field and they can be polarized via a magnetic field [63]. The ME effect is the induction of electrical polarization by the application of magnetic field or magnetization by applying electric field [32,63]. The ME effect can be the linear and nonlinear coupling between the electrical and magnetic order parameters, as described by Landau free energy expression [1,2]

(1) $$\begin{array}{ll} G\left(E,\;H\right)= & G_{0}-P_{i}{}^{s}E_{i}-\;M_{i}{}^{s}H_{i}-\frac{1}{2}\varepsilon_{0}\varepsilon_{ij}E_{i}E_{j}-\frac{1}{2}\mu_{0}\mu_{ij}H_{i}H_{j}-\alpha_{ij}E_{i}H_{j}-\frac{1}{2}\beta_{ijk}E_{i}H_{j}H_{k}\\ & \;\;\;-\frac{1}{2}\gamma_{ijk}H_{i}E_{j}E_{k}-\;\frac{1}{2}\delta_{ijkl}E_{i}E_{j}H_{k}H_{l}-\cdots \end{array}$$

Here, *E* is electric field, *P*^S^ is spontaneous polarization, *H* is magnetic field, *M*^S^ is spontaneous magnetization, *μ* is magnetic susceptibility, *ε* is electric susceptibility, *α* is linear ME coefficient, and *β*, *γ*, and *δ* are higher order of ME coefficients. The coupling coefficients are tensor, as *E* and *H* are vectors [1,2]. The ME effect in composite systems is a product property, which arises because of the cross coupling between the electrical and magnetic order parameters of the FE and magnetic phases [64]. The separate ferroelectric and magnetic phase do not show a ME effect, but the hybrid composite system consisting of both the phases exhibits large ME coupling [65]. There are generally two types of ME coupling: (a) direct ME coupling and (b) converse ME coupling, depending on the elastic interactions and applied electric or magnetic field [32,46].

(2) $$(a)\;\mathrm{Direct}\;\mathrm{ME}\;\mathrm{coupling}\;=\;\frac{\mathrm{Magnetic}}{\mathrm{Mechanical}}\;\times \;\frac{\mathrm{Mechanical}}{\mathrm{Electrical}}$$

(3) $$(b)\;\mathrm{Converse}\;\mathrm{ME}\;\mathrm{coupling}\;=\;\frac{\mathrm{Electrical}}{\mathrm{Mechanical}}\;\times \;\frac{\mathrm{Mechanical}}{\mathrm{Magnetic}}$$

### 2.1. Direct ME Coupling

When a composite system is subjected to an applied magnetic field, then mechanical strain develops in the magnetic phase via the piezomagnetic/magnetostrictive (magnetic/mechanical) effect. It may be noted that piezo-magnetism is the linear function of change of strain as a function of applied *H* or the change of magnetization as a function of applied stress, whereas the magnetostrictive effect is the change of strain as a quadratic function of applied *H* [1,2]. The developed strain in the magnetic phase transfers to the FE phase and modifies the FE and other electrical properties via the electro-strictive/piezoelectric (mechanical/electrical) effect. It should be noted that the piezoelectric effect is a linear change of polarization as a function of applied stress or change in strain as a function of *E*, whereas electrostriction is a quadradic change of strain with applied *E* [1,2]. This coupling is called direct ME coupling [17,32]. In this case, the coupling takes place via mechanical strain transmission (which is an elastic interaction) between the FE and magnetic phases [2]. In most of the cases, direct ME coupling takes place via strain that is induced by the magnetic field. There are many reports in literature showing the evidence of direct ME coupling in composites, whereas there are very few reports on converse ME coupling.

**Figure 3:**
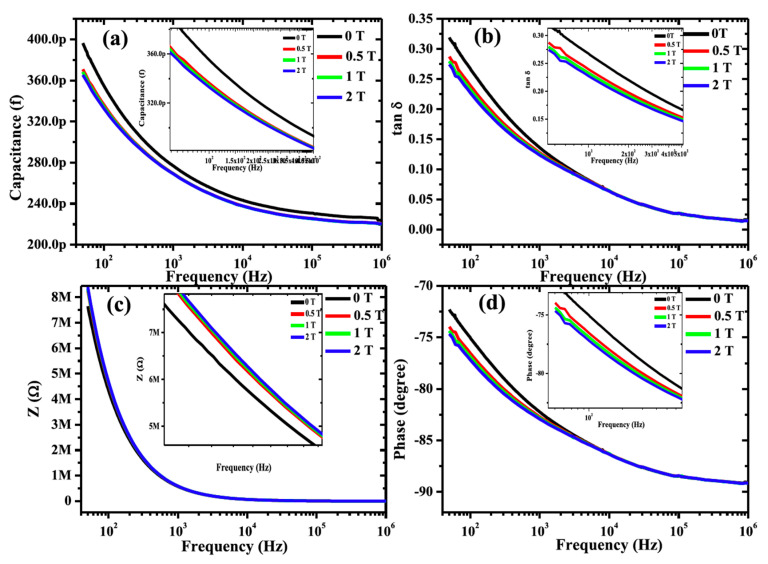
Frequency dependence of (**a**) capacitance, (**b**) loss, (**c**) impedance, and (**d**) phase of PN2 composite at different static magnetic field. Figure reproduced with permission from [66].

The evidence of direct ME coupling in (1 − x)Pb(Fe_0.5_Nb_0.5_)O_3__−_xNi_0.65_Zn_0.35_Fe_2_O_4_ (x = 0.2) (abbreviated as PN2) ceramic composites has been demonstrated and is shown in Figure 3 [66]. Direct ME coupling in multiferroic materials can be verified by probing the variation of dielectric parameters with the application of different static magnetic field [66]. Frequency dependence of the dielectric parameters, such as capacitance (*C*p), loss tangent (tanδ), impedance (*Z*), and phase (*θ*) measurements at different static magnetic field (0 ≤H≤2 T), were performed at room temperature (Figure 3). A significant and systematic decrease in the parallel capacitance, loss tangent, and phase angle were observed, whereas the impedance was found to increase with an increase in the magnetic field. The variation of the dielectric parameters is observed to be larger at 0.5 T when compared to other applied magnetic fields, which might be due to the *H*-dependence of magnetostriction behavior of these samples. It was also found that a change of the dielectric parameters with varying magnetic field is higher at lower frequencies (100–10^4^ Hz), because of the existence of all types of polarization dynamics at low frequencies. The tuning of dielectric parameters with the variation of magnetic field confirms the existence of strong direct ME coupling in theses composite systems at RT [66]. The quantitative and qualitative discussion on direct ME coupling is described in more details in the magnetodielectric coupling section.

### 2.2. Converse ME Coupling

When a composite system is subjected to an external electric field, it induces strain in the FE phase through inverse electrostrictive/piezoelectric (electrical/mechanical) effect. This induced strain in FE phase transfers to the magnetic phase and via the inverse magneto-strictive/piezomagnetic (mechanical/magnetic) effect, it can switch and tune the magnetization. This coupling is termed converse ME coupling [32,66]. This coupling takes place via an elastic interaction through the inverse electrostrictive/piezoelectric and magneto-strictive/piezomagnetic effect between the FE and magnetic phases. There are several mechanisms that are involved in converse ME coupling, such as: (i) strain, (ii) variation of spin-polarized charge densities, (iii) modulation of interfacial oxidation, and (iv) coupling due to spin exchange. All of these mechanisms take place by applying electric field and tune the magnetization [34,50,54].

**Figure 4:**
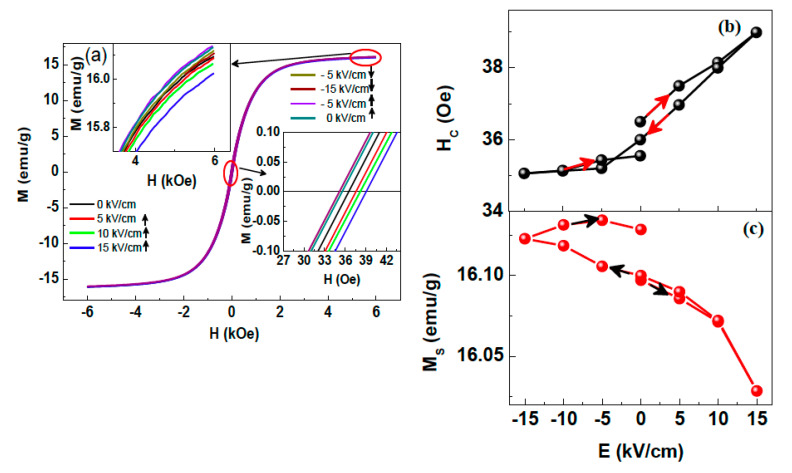
(**a**) *M*-*H* loops at different external applied *E*-fields at RT (Inset: magnified *M*-*H* loop at high and low magnetic fields. (**b**) Variation of coercive field (*H*_c_) (**c**) saturation magnetization (*M*_s_) with external applied *E*-fields of PN2 composite (Arrows indicate the direction of *E*-field). Figure reproduced with permission from [66].

The existence of converse ME coupling at RT in PN2 ceramic composites is demonstrated and shown in Figure 4 [66]. In order to verify the existence of converse ME coupling in PN2 composite, the *M*-*H* hysteresis loops measurements have been carried out at different dc *E*-fields (Figure 4). A systematic and notable change in *H*_c_ and *M*_s_ by varying *E*-field was observed, and these magnetic orderings were tunable by changing the direction of *E*-field (Figure 4b,c). Hence, converse ME coupling has been demonstrated in the PN2 composite system [66]. Sanchez et al. also investigated the evidence of converse ME coupling in a single phase multiferroic PZTFT bulk ceramic sample [67].

The composites should exhibit distinct and well-defined interfaces among the FE and magnetic phases in order to increase the magnitude of ME coupling. Highly asymmetric epitaxial growth of multilayered structurers of alternative magnetic and FE layers with sharp interfaces is one of the best ways to achieve strong ME coupling as perfect strain transmission will take place among the sharp interfaces. The lower thickness of the magnetic layers when compared to FE layers in these heterostructures will minimize dielectric loss and leakage currents. The chemical reaction between the FE and magnetic materials will decrease the magnitude of ME coupling. While choosing the FE and magnetic materials, special attention should be given in order to avoid chemical reactions between them. ME composites with relaxor ferroelectrics having high piezoelectric coefficient as FE candidate and magnetic materials with high resistivity along with *T*_C_ above RT should be investigated.

## 3. Mechanisms of ME Coupling

ME coupling in composite structures depends on the interplay among the charge, spin, lattice, and orbital degrees of freedom at the interphase boundaries (more accurately across the interfaces). There are several mechanisms that are involved in the origin of ME coupling [50].

### 3.1. Strain Mediated ME Coupling

The ME coupling in most of the composite systems arises due to elastic interactions among the FE and magnetic phases [22,50]. In ME composites, ME coupling takes place as FE and magnetic domains are coupled via their secondary (ferro/magneto) elastic domains because of electrostriction and magnetostriction. The evolution of domains in composites is very sensitive to the microstructure, such as: size, shape, orientation, and distribution of the grains of both the phases [32,33]. The strain mediated ME coupling has already been discussed in introduction, along with direct and converse ME effect sections. The physical properties, such as transition temperature (*T*_C_), ferroelectric, magnetic, and ME properties, can be significantly improved by appropriate strain engineering. Strain can be developed in materials via lattice mismatched growth, defects, doping, and by applying external electric and magnetic field. The strain developed in materials can modify the crystal structure, ionic distribution, clustering of atoms, oxygen coordination, vacancies, microstructures, compositions, etc. [17,32,34,47]. Epitaxial strain generated because of lattice mismatched growth is a nondestructive approach for significantly enhancing the functionalities [68]. The strain generated at multiple interfaces of multilayer oxide heterostructures and superlattices can give rise to new quantum states and enhanced physical functionalities [65,68]. Because strain plays an important role in determining ME coupling in composite structures, FE materials having high electro-strictive/piezoelectric coefficients and magnetic candidates having high piezomagnetism/magnetostriction along with high resistivities are necessary for designing different composite architectures to produce large ME coupling [12,32,50].

In ME composite structures, the strain that developed in the FE phase by applying an electric field and its transmission to the magnetic phase depend on the nature of FE phase, FE domain and domain wall, orientation of polarization and resistivity [50,69]. The direction of spins of the magnetic phase gives rise to magnetic domains and domain walls, non-uniform magnetic anisotropy, residual strain, and pinning area having lower magnetization [34,50]. The strain that is generated in the magnetic phase via magnetic field is not exactly converse to that of strain generated by *E*-filed. The ME coupling in composites are substantially reduced by defects, residual strains, grain boundaries, clamping effects, dislocations, and voids/pores [11,17]. Good strain transmission between the FE and magnetic phase is necessary in order to observe large ME coupling in composite structures [32,65]. However, well controlled strain generation and transmission in the composite structures is not trivial to obtain.

### 3.2. Charge Mediated ME Coupling

This charge mediated ME coupling is generally observed in 2-2 type (layered heterostructures) composite nanostructures that contain ultrathin ferromagnetic films. By applying an electric field, bound charges at the FE interface accumulate, which modifies the charge density of the magnetic layer via charge screening [34,50]. This mechanism enables the significant modulation of the carrier density in oxides, where the carrier density is generally ~10^21^ cm^−3^. This approach is reversible and nonvolatile without any structural distortion. By modulating charge carrier density, the FE, magnetic, and transport properties of these composite structures can be tailored via electron correlations, control of magnetism, and orbital state [34,54]. Charge screening in the magnetic component causes the modification in spin structure at the Fermi level compared to the equilibrium state. By applying an electric field across the dielectric layer instead of a vacuum barrier, the ME coupling can be enhanced because of the higher dielectric permittivity of the dielectric material. In addition, chemical bonding at the interfaces also plays an important role in the ME coupling [34,50]. Theoretically, it is predicted that, in Fe/BaTiO_3_ heterostructures, a large ME coupling arises because of the modification of the chemical bonding at the interface [70,71,72,73,74]. The modification of charge density at interfaces also significantly changes the magnetic anisotropy of the magnetic material due to spin–orbit coupling via the reconstruction of the electron occupancy of various d-orbitals. The electrical and magnetic properties of strongly correlated oxides are very sensitive to charge density; hence, both of the properties can be electrostatically tuned [34,70,71,72,73,74].

**Figure 5:**
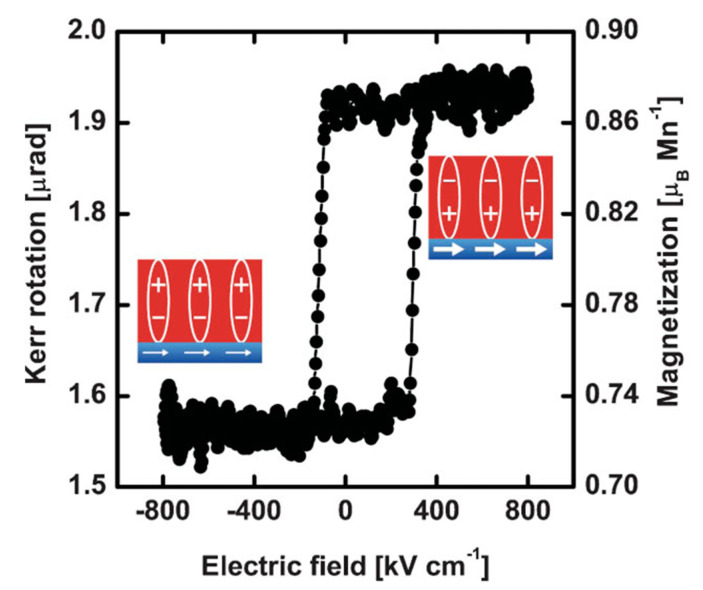
ME hysteresis loop at 100 K demonstrating the applied electric field dependence on the magnetic behavior of the PZT/La_0.7_Sr_0.3_MnO_3_ (LSMO) heterostructure. The two magnetization values indicating the modulation of the magnetization values of LSMO layer (Insets: the FE and magnetic states of the PZT and LSMO layers respectively. The size of the white arrows implies the amplitude of magnetization qualitatively). Figure reproduced with permission from [54].

Molegraaf et al. demonstrated electric field modulation of magnetization through the charge mediated ME coupling mechanism in Pb(Zr_0.2_Ti_0.8_O_3_) (250 nm)/La_0.8_Sr_0.2_MnO_3_ (4 nm) bilayer heterostructures grown on (100) oriented SrTiO_3_ substrate (Figure 5) [54]. Figure 5 demonstrates the electric field dependence of the magnetic behavior of the heterostructure, which shows a notable change of the magnetization of LSMO, when the polarization of PZT switches. This *M*–*E* curve shows coupling between the FE and magnetic ground states and exhibits a magnetic hysteretic behavior by varying *E*-field. In this case, the coupling between the polarization and magnetization arises via charge density modulation and is directly related to the surface bound charge of the gate oxide. They observed a large ME coupling coefficient of ~0.8 × 10^−3^ V^−1^ (Oe·cm), which is significantly higher than well-known single-phase multiferroic materials [54]. The magnetic *T_C_* of LSMO is found to increase by ~20 K, because of significant ME coupling in these heterostructures. The switching and tuning of magnetism of the LSMO layer with the variation of applied *E*-field is attributed to electronic charge density modulation based ME coupling [34,54].

### 3.3. ME Coupling Due to Spin Exchange

The exchange bias (EB) effect arises because of the coupling between the spins of a ferromagnetic (FM) material and the spins (uncompensated interfacial) of an antiferromagnetic (AFM) materials [73]. This effect is characteristic of FM/AFM interfaces. The spin structure and direction of EB field at the interface can be modified and controlled by switching the FE polarization by applying external *E*-field. *E*-field control of EB has been experimentally investigated in MF/FM bilayer heterostructures having YMnO_3_, LuMnO_3_, BiFeO_3_ as MF candidates that exhibit FE and AFM behavior [74,75,76]. In (1 − x)BiFeO_3−_xFe_3_O_4_ ME composite thin films, *E*-field control of EB has been achieved at room temperature [61]. Laukhin et al. demonstrated the *E*-field control of EB coupling and switching of magnetization in NiFe/YMnO_3_/Pt multiferroic heterostructure [77]. The direct and reversible *E*-field tuning of EB in BiFeO_3_ (BFO)/La_0.7_Sr_0.3_MnO_3_ (LSMO) ME heterostructures have been experimentally and theoretically investigated by different research groups [34,55,56,74,78]. In this case, the application of an *E*-field causes ionic displacements in BFO via FE switching and changes the interatomic distance among the Fe and Mn and cations at the interface, which gives rise to a change in the EB coupling [34]. Because BFO exhibits ferroelasticity, the contribution of strain cannot be ruled out. A first principle study demonstrated that the FE polarization (*P*), canted magnetization (*M*c = *M*_Fe1_ + *M*_Fe2_) and the AFM vector (L = *M*_Fe1_ − *M*_Fe2_), where *M*Fe_1_ and *M*Fe_2_ are the magnetizations of sublattices are mutually perpendicular [50,79]. Hence, polarization switching by an external applied *E*-field can tune and switch the magnetization, which, in turn, modulates the EB interaction between the canted magnetization in BFO and the magnetization of different FM materials [34,80]. This explanation has been used to explain the experimental investigation of room temperature *E*-field modulated 180° magnetization switching in a Co_0.9_Fe_0.1_/BFO heterostructures [81]. However, more theoretically guided experimental investigations are required in order to unravel the exact reasons of exchange bias mediated ME coupling. EB effects in ME nanostructures make them suitable for new memory technologies, such as magnetoelectric RAM (MERAM) [18,40,42,61].

The coupling between the polarization and magnetization need to be probed by advanced scanning probe microscopy (piezoresponse force microscopy (PFM) and magnetic force microscopy (MFM)) experiments by observing the dynamics of FE domains by applying magnetic field and magnetic domains by applying electric field in order to unravel the exact mechanisms of ME coupling at the nanoscale. Designing new types of domain structures, such as magnetic skyrmions and polar vortices in composite structures, in order to observe new types of ME coupling is worthy of investigations.

## 4. ME Coupling Coefficient

ME coupling indicates the tuning of electrical polarization by applying external magnetic field and the modification of magnetization by applying the external electric field [2,10]. The effect can be represented by ME coupling coefficient (α) [1].

Electrically induced ME coupling describes the change in magnetic induction (*B*) of the material by applying an *E*-field [1,2,82,83].

(4) $$\alpha_{ij}^{E}=\;\left(\frac{\partial B_{i}}{\partial E_{j}}\right)$$

Magnetically coupling describes the change in the electrical polarization (*P*) by applying a magnetic field (*H*):

(5) $$\alpha_{ij}^{H}=\;\left(\frac{\partial P_{i}}{\partial H_{j}}\right)$$

*E* = (*V*/*t*); where *V*-voltage and *t*-thickness of the sample.

Hence, magnetically induced ME effect can be represented as

(6) $$\alpha_{ij}^{H}=\;\left(\frac{\partial P_{i}}{\partial H_{j}}\right)=\;\varepsilon_{0}\varepsilon_{ii}\left(\frac{\partial E_{i}}{\partial H_{j}}\right)=\;\frac{\varepsilon_{0\;}\varepsilon_{r}}{t}\;\left(\frac{\partial V}{\partial H}\right)=\;\varepsilon_{0}\varepsilon_{r}\alpha_{V}^{H}$$

where $\alpha_{V}^{H}$—magnetically induced ME voltage coefficient (MEVC)

(7) $$\alpha_{V}^{H}=\left(\frac{\partial E}{\partial H}\right)=\;\frac{1}{t}\;\left(\frac{\partial V}{\partial H}\right)$$

The MEVC is the most common parameter for the analysis of experimental results.

The relation between ME coupling coefficient ($\alpha^{H}$) and MEVC ($\alpha_{V}^{H}$) is as follows

(8) $$\alpha^{H}=\;\varepsilon_{0}\varepsilon_{r}\alpha_{V}^{H}$$

Both $\alpha^{H}$ and $\alpha^{E}$ are expressed as [s/m] in SI unit, whereas MEVC is expressed in [V/A] in SI units and [V/(cm·Oe)] in CGS units [82,83].

$$\alpha^{H}\;\propto d.\;q$$

where *d* is the piezoelectric coefficient and *q* is the piezomagnetic coefficient: *q* = *dλ*/*dH* (*λ* is the magnetostriction) of the ME material [82].

ME materials with high $\alpha_{V}^{H}$ values at RT are suitable for practical device applications, which can be suitably controlled by magnetic and electric field. Besides the conventional architectures (such as 0-3, 2-2, and 1-3), some hybrid composite architectures with suitable microstructural designs also need to be explored in order to observe large ME coupling at RT.

Room-temperature $\alpha_{V}^{H}$ value of various bulk and nanostructured magnetoelectric composites are presented in Table 1 and Table 2, respectively.

## 5. Magnetodielectric Coupling

The existence of coupling between the ferroelectric and magnetic order parameters have been established in a wide range of multiferroic materials using dielectric spectroscopy in the presence of an applied magnetic field [11,125,126]. In ME materials, magnetic ordering is coupled to the electric polarization; hence, to the dielectric parameters. The existence of magnetodielectric (MD) coupling can be confirmed if multiferroic materials fulfil at least one out of these two criteria: (i) significant and systematic change in dielectric permittivity (capacitance) by increasing or decreasing the applied magnetic field and (ii) the appearance of an anomaly or a change in the temperature dependent dielectric spectra around the magnetic transition temperature (*T_C_* or *T_N_*) [17,125].

The MD coupling can be quantitatively expressed by magnetocapacitance (*MC* %), magneto-impedance (*MI* %), magneto-loss (*ML* %), magneto-phase (*MP* %) and it can be expressed by the formulae below [11,66,125,126,127,128,129].

(9) $$MC\;\%=\;\frac{C_{p}\;\left(H,T\right)-C_{P}\;\left(0,T\right)}{C_{p}\;\left(0,T\right)}\;\times \;100$$

(10) $$MI\;\%=\;\frac{Z\;\left(H,T\right)-Z\;\left(0,T\right)}{Z\;\left(0,T\right)}\;\times \;100$$

(11) $$ML\;\%=\;\frac{\tan\delta \;\left(H,T\right)-\tan\delta \;\left(0,T\right)}{Z\;\left(0,T\right)}\;\times 100$$

(12) $$MP\;\%=\;\frac{\theta \;\left(H,T\right)-\theta \;\left(0,T\right)}{\theta \;\left(0,T\right)}\;\times \;100$$

Here, *C_p_* is the capacitance, *Z* is the impedance, tanδ is the loss tangent, and *θ* is the phase of the ME material.

The existence of ME coupling in different single-phase and composite multiferroics systems have been confirmed by (*MC* %), (*MI* %), (*ML* %), and (*MP* %) measurements [17,24,25,66].

Fulfilling these two criteria is often not enough to conclude the presence of intrinsic ME coupling in MF materials [22,126]. Because strong MD coupling can also be realized via the combination of the Maxwell–Wagner (M–W) effect and magnetoresistance (MR) in materials, which is not related to true ME coupling [125,126,128]. Except ME coupling, other extrinsic effects such as magneto-resistive artifacts can also exhibit a MC or MD effect. Hence, it is important to note that ME coupling might exhibit MD coupling, but the converse is not always true [22,125]. Several materials do not exhibit multiferroism, but show MD coupling. When one measures the dielectric properties of MF materials, metal-insulator-metal capacitor structures are fabricated in order to apply an ac electric field across it. Generally, the work function of the dielectric materials and the electrodes are not identical; hence, band bending develops near the dielectric- electrode interface, which can give rise to charge injection from the electrode to dielectric material or vice versa [17,22,127,128]. In either case, the formation of a layer takes place near the interface having dissimilar charge density; hence, dissimilar resistivity than the core. If the dielectric material is not highly resistive, then the electric field will be mostly dropped in the interfacial region instead in the core of the dielectric; this leads to very high dielectric permittivity. This type of effect also occurs at grain-grain boundaries in bulk ceramics and at the interfaces of the high and low resistive superlattices [23,126]. The different nature of the electrode–dielectric interfaces in superlattice and grain–grain boundary in ceramics can be expressed by the M–W capacitor model [125,126,128]. The M–W capacitor model has been demonstrated in detail elsewhere. According to this model, the real and imaginary part of dielectric permittivity can be expressed, as follows [126,128]

(13) $$\varepsilon^{\prime }\left(\omega \right)=\frac{1}{C_{0}\left(R_{i}+R_{b}\right)}\;\frac{\tau_{i}+\tau_{b}-\tau +\omega^{2}\tau_{i}\tau_{b}\tau }{1+\omega^{2}\tau^{2}}$$

(14) $$\varepsilon^{\prime \prime }\left(\omega \right)=\frac{1}{\omega C_{0}\left(R_{i}+R_{b}\right)}\;\frac{1-\omega^{2}\tau_{i}\tau_{b}+\omega^{2}\tau \;(\tau_{i}+\tau_{b})}{1+\omega^{2}\tau^{2}}$$

where subscripts *i* stands for interfacial type layer, *b* stands for bulk-type layer, ω stands for ac frequency, *C* stands for capacitance, *R* stands for resistance and $\tau_{b}=\;C_{b}R_{b}$, $\tau_{i}=\;C_{i}R_{i}$, $\tau =\left(\tau_{i}R_{b}+\tau_{b}R_{i}\right)/\left(R_{b}+R_{i}\right)$, $C_{0}=\left(\varepsilon_{0}A\right)/t$, here $\varepsilon_{0}$ is free space permittivity, *A* is capacitor area, and *t* is the thickness of the sample.

The above equations indicate that, if any resistance (*R_i_* or *R_b_*) changes by applying magnetic field, then one will observe a change in the measured dielectric permittivity. M–W effect combined with the MR can provide an MD or MC effect, which are not necessarily an indication of true ME coupling [125,126,127]. The existence of intrinsic (true) ME coupling in a MF composite has been demonstrated by computing the real and imaginary part of impedance, from the dielectric parameters (*C_p_*, *Z*, tanδ, and *θ*). The detection of true ME coupling in (1 − x)Pb(Fe_0.5_Nb_0.5_)O_3__−_xCo_0.65_Zn_0.35_Fe_2_O_4_ (x = 0.2) (abbreviated as PC2) ceramic composite system is discussed in detail from the perspective of detailed dielectric spectroscopy measurements [22].

The occurrence of extrinsic M–W and MR effects due to interfacial contributions in MC, particularly in MF materials, can be separated from the intrinsic contributions by impedance analysis. The bulk capacitance (*C_b_*) and bulk resistance (*R_b_*) can be extracted from impedance analysis, which are intrinsic in nature without any contributions from MR and M–W effects [22].

**Figure 6:**
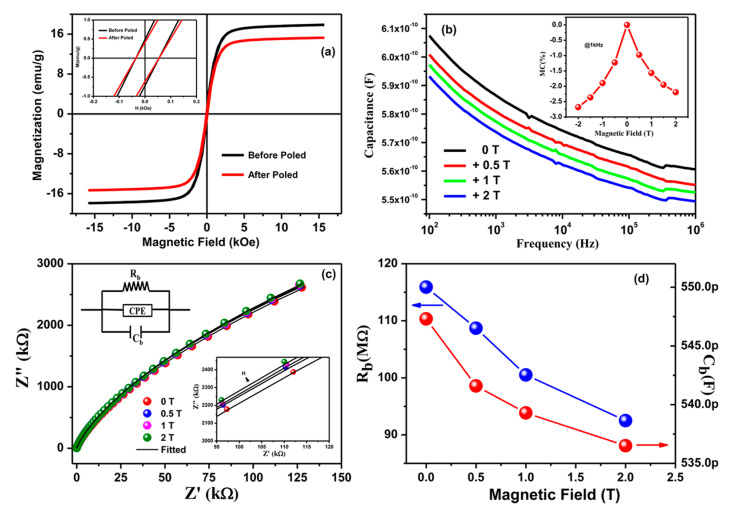
(**a**) Magnetic Field dependence of magnetization (*M*(*H*)) loops with and without electrical poling. (**b**) Capacitance as a function of frequency at different static *H*. (**c**) *Z*’ (real part of impedance) vs. *Z*’’ (imaginary part of impedance) plot at different *H* (solid line indicate the fitted data); the equivalent circuit used for fitting (inset). (**d**) Variation of bulk capacitance (*C_b_*) and bulk resistance (*R_b_*) at different *H*. Figure reproduced with permission from [22].

First, the existence of ME coupling was verified by measuring the *M*–*H* loops of the sample without and with electrical poling by applying electric field of ~2 kV/cm at RT (Figure 6a) [22]. The saturation magnetization (*M_s_*) and remnant magnetization (*M_r_*) were found to decrease after electrical poling. The tuning of magnetization by electrical poling indicated the coupled electrical and magnetic orderings in these composites. Subsequently, the MD coupling was tested by measuring the capacitance as a function of frequency at different static *H* (Figure 6b) at RT. The systematic and notable decrease of capacitance with increasing *H* over the entire frequency range implied the existence of significant MD coupling in these composites. The *MC* % has been calculated for both positive and negative *H* at 1 kHz and presented in the inset of Figure 6b. The *MC* % was found to be *~*−2.19 and ~−2.68 for applied *H* of + 2 T and −2 T, respectively [22]. The negative sign depends on the coupling constant and the spin interactions among the negative spins of the surrounding spins [24]. It was discussed earlier that large MD coupling in samples can also arise due to extrinsic effects. In order to prove the observed significant MD coupling in PC2 composite was intrinsic in nature, the *Z*’ (real part of impedance) and *Z*’’ (imaginary part of impedance) were calculated and plotted in Figure 6c at different *H*. The Nyquist plots (*Z*’ vs. *Z*’’) were fitted while using the equivalent circuit shown in the inset of Figure 6c. The calculated/fitted values were found to closely match the experimental values. The values of bulk resistance (*R_b_*) and bulk capacitance (*C_b_*) have been extracted from the fitting and also plotted as a function of static *H* (Figure 6d). A systematic decrease of *R_b_* and *C_b_* with increasing of *H* was determined. This variation of *R_b_* and *C_b_* by the application of *H* confirmed the intrinsic nature of ME coupling, as *C_b_* and *R_b_* are free from extrinsic effects. The confirmation of intrinsic nature of ME coupling via dielectric spectroscopy measurements has also been reported in single-phase multiferroic materials [127].

The existence of intrinsic ME coupling via MD measurements needs to be carefully investigated, as extrinsic effects, such as M–W and MR effects, can significantly contribute to the magnitude of MD coupling.

## 6. Nature of ME Coupling

Despite many years of research on multiferroic materials, the nature of the interaction between the FE and magnetic order parameters (ME coupling) are still not completely understood [23]. The nature of ME coupling i.e., the coupling between the polarization (*P*) and magnetization (*M*) might be bilinear (*P*–*M*), linear-quadratic (*P*–*M*^2^), quadratic-linear (*P*^2^–*M*), biquadratic (*P*^2^-*M*^2^), linear–cubic (*P*–*M*^3^), cubic-linear (*P*^3^–*M*), or higher order terms [22]. Among the possible natures of ME coupling, only *P*–*M* is direct coupling, while other coupling takes place via elastic interactions. The detection of the nature of ME coupling is very important and challenging [22]. The nature of ME coupling in PC2 multiferroic composites has been probed utilizing Landau’s free energy that is expressed in the following equation [22,23].

(15) $$G=\;\overset{\infty }{\underset{i=1}{\sum }}A\left(i\right)P^{2i}-E.P+\overset{\infty }{\underset{j=1}{\sum }}B\left(j\right)M^{2j}-H.M+\overset{\infty }{\underset{k=1}{\sum }}C\left(k\right)\varepsilon^{2k}-S.\varepsilon +\;\overset{\infty }{\underset{i,j,k=0}{\sum }}D\left(i,j,k\right)P^{i}M^{j}\varepsilon^{k}\;$$

Here, *E* is electric field, *P* is polarization, *M* is magnetization, *H* is magnetic field, ε is strain, *S* is stress, and *A*, *B*, and *C* are constants.

The first two terms in equation represent the energy expansion of *P* and the work done by *E*, the third and fourth terms represent energy expansion of *M* and the work done by *H*, the fourth and fifth terms represent energy expansion of ε and the work done by *S*, whereas the last term indicates the combination of all probable coupling among *P*, *M*, and ε.

Under equilibrium conditions $\frac{dG}{dP}=0$, the inverse of susceptibility ($\chi^{-1}=\frac{dE}{dP})$ can be expressed as

(16) $$\chi^{-1}=\overset{\infty }{\underset{i=1}{\sum }}A\left(i\right)2i\left(2i-1\right)P^{2i-2}+\overset{\infty }{\underset{i,j,k=0}{\sum }}D\left(i,j,k\right)i\left(i-1\right)P^{i-2}M^{j}\varepsilon^{k}\;$$

Here, $\chi^{-1}$ can be scaled as the inverse of capacitance (*C*^−1^), where capacitance can be directly measured.

As in ME materials, the FE and magnetic orderings are both coupled; hence, $\chi^{-1}$ can be varied as a function of *H*, by the following equation

(17) $$\frac{d\chi^{-1}}{dH}=\;\overset{\infty }{\underset{i,j,k=0}{\sum }}D\left(i,j,k\right)i\left(i-1\right)P^{i-2}j\;\frac{dM}{dH}M^{j-1}\varepsilon^{k}\;$$

By correlating the magnetic field derivative of inverse of susceptibility $\left(\frac{d\chi^{-1}}{dH}\right)$ with magnetic field (*H*), the nature of coupling can be directly probed from the Landau’s free energy equation. Here, $\chi^{-1}$ can be scaled as the inverse of capacitance (*C*^−1^), where capacitance can be measured directly.

If the only active coupling is bilinear (*P*–*M*), then *(i − 1)* term will be zero, then $\left(\frac{d\chi^{-1}}{dH}\right)$ will be zero for all applied *H*, if the only active coupling is quadratic-linear (*P*^2^–*M*), then $\left(\frac{d\chi^{-1}}{dH}\right)$ will be proportional to $\frac{dM}{dH}$, and if the coupling is dominated by quadratic term (*P*^2^–*M*^2^), then $\left(\frac{d\chi^{-1}}{dH}\right)$ should be really proportional to $M\left(\frac{dM}{dH}\right)$ [22].

**Figure 7:**
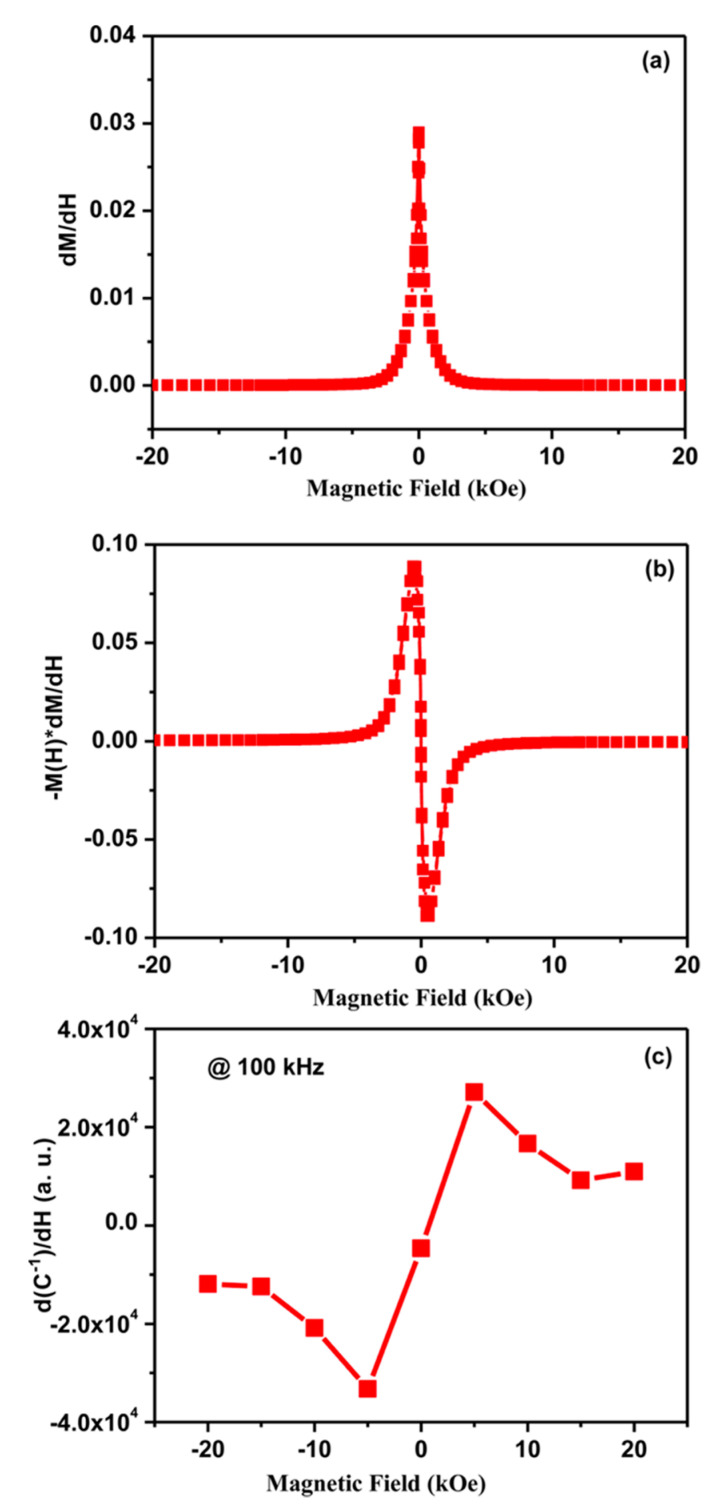
Magnetic field dependence of (**a**) *dM*/*dH* (**b**) M(*dM*/*dH*), and (**c**) *dC*^−1^/*dH* of PC2 composite. Figure reproduced with permission from [22].

To detect the behavior of coupling in PC2 composite, $\left(\frac{dM}{dH}\right)$ and $-M\left(\frac{dM}{dH}\right)$ as a function of applied *H* were graphed and are shown in Figure 7a,b. For the comparison of $\left(\frac{dM}{dH}\right)$ and $-M\left(\frac{dM}{dH}\right)$ as a function of applied *H* with $\left(\frac{d\chi^{-1}}{dH}\right)$, $\left(\frac{dC^{-1}}{dH}\right)$ as a function of *H* was plotted, where *C* is a experimentally measured quantity (Figure 7c). It can be clearly seen from the Figure that the behavior of $\left(\frac{dC^{-1}}{dH}\right)$ curve is quite identical to that of the $-M\left(\frac{dM}{dH}\right)$ curve, which implies that biquadratic (*P*^2^–*M*^2^) coupling was dominant in these MF composite systems. The presence of biquadratic coupling in these composites indicates that the ME effect was not the direct coupling between *P* and *M*, but occurs indirectly through a ferroelastic effect. Here, electrostriction and magnetostriction are responsible for the ME coupling, which couple to the square of the ordering parameter [22].

The nature of ME coupling investigated via bulk dielectric and magnetic measurements along with Landau free energy expression should be corroborated by probing the coupling of FE and magnetic domains at the nanoscale. Moreover, understanding the new physics of ME coupling and controlling and/or switching the FE and magnetic domains by applying ultrafast stimuli (such as optical, electrical, and acoustic) in composite structures is worth exploring.

## 7. Summary and Future Directions

ME composite structures, which integrate FE and magnetic materials, have drawn intense interest due to the interesting physics of coupling of polarization and magnetization, as well as their applications in various novel room temperature multifunctional devices. ME composites possess large polarization, magnetization, and ME coupling at RT. For practical applications, this requires that the FE and magnetic ordering temperatures (*T*_C_) of ME composites occur well above RT. ME coupling and other physical properties in these composites can be suitably enhanced by the proper design of various types of connectivity of the FE and magnetic phases. Additionally, selecting appropriate FE and magnetic materials, suitable microstructural design, synthesis of phase pure structures, and good epitaxial growth are all critical for strong ME coupling. In ME composites, the coupling of FE and magnetic order parameters takes place via strain, charge coupling, and spin exchange (exchange bias) between the phases. Biquadratic (*P*^2^–*M*^2^) coupling typically dominates in these ME composite structures.

The investigations on ME composites reported so far reveal promising outcomes with significant progress along with the demonstration of some prototype devices; however, still many unsolved issues need to be addressed towards real device applications.

- Discovery of new lead-free room temperature ME materials guided by theory having strong cross coupling between magnetization and polarization, low leakage current, and high remanent magnetization should be given the top priority.
- Elucidate the effect of microstructure on ME coupling.
- Explore new FE and magnetic domain structures, such as magnetic skyrmions and polar vortices, as this might lead to different types of ME coupling.
- Examine the behavior of ferroic order parameters under the application ultrafast stimuli (optical, acoustic, and electrical).
- Investigate the role of MF orderings on the emergence of different quantum phenomena as this could open completely new directions.
- Maintain the thermal stability at RT of strongly coupled magnetic and FE orderings at 10-nm length scale is one of the most important requirements for emerging technologies.
- Advanced characterization tools are required in order to study multiferroic and ME properties at the nanometer and atomic lengthscale.
- The FE and/or ME switching voltage should be ≤100 mV for realization of ultra-low power devices.
- Successful integration on-chip, compatibility with different process technologies, and optimizing the ME device performance are all required for ME materials to penetrate practical device applications.

It is evident that the field of ME materials will make significant breakthroughs in the near future and, thus, this family of materials is an intriguing area of research, which can lead to a plethora of important applications.

## Figures and Tables

**Table 1:** Room-temperature α_HV_ value of bulk magnetoelectric composites.

Bulk	*H*_DC_ (Oe)	f_AC_ (Hz)	$\alpha_{V}^{H}(\mathrm{mV}\;{\mathrm{cm}}^{-1}\;{\mathrm{Oe}}^{-1})$	Note	Reference
0.9Pb(Zr_0.52_Ti_0.48_)O_3_-0.1NiFe_1.9_Mn_0.1_O_4_	100	1000	140	0-3 composite, Longitudinal	[84]
PZT-20 wt%NiCo_0.02_Cu_0.02_Mn_0.1_Fe_1.8_O_4_	1250	100	115	0-3 composite, Longitudinal	[85]
P(VDF-TrFE)-CoFe_2_O_4_	2000	5000	40	0-3 composite, Longitudinal	[86]
Ni_0.8_Zn_0.2_Fe_2_O_4_-0.41 vol% PZT	250	100	45	0-3 composite, Transverse	[87]
0.68Pb(Zr_0.57_Ti_0.43_)O_3_-0.32NiFe_2_O_4_	1000	1000	80	0-3 composite, Longitudinal	[88]
0.62BTO-0.38CoFe_2_O_4_ (1.5 wt% excess TiO_2_	560	1000	130	3-3 composite, Longitudinal	[89]
PMN-PT-NiFe_2_O_4_	2400	0	10.43	0-3 composite, Transverse	[90]
0.6BTO-0.4Ni(Co,Mn)Fe_2_O_4_	500	1000	81.7	3-3 composite, Longitudinal	[91]
Metglas/PVDF	2	20	21,460	2-2 composite, Transverse	[92]
Metglas/PMN-PT (fiber)/Metglas	8	1000	52000	2-1 composite, Longitudinal	[93]
Metglas (fiber)/PMN-PT (fiber)/Metglas (fiber)	2	1000 (23000)	29310 (7,000,000)	1-1 composite, Transverse	[94]
Metglas/PVDF/Metglas	8	1000	7200	2-2 composite, Transverse	[95]
Metglas/PMT	-	1000	63.3	2-2 composite, Transverse	[96]
Metglas/PZT (fiber)/Metglas	-	1000 (33,700)	12,000 (380,000)	2-1 composite, Longitudinal	[97]
FeBSiC/PZN-PT (fiber)/FeBSiC	2	1000 (20,000)	10500 (400,000)	2-1 composite, Transverse	[98]
FeBSiC/PZT (fiber)/FeBSiC	4	1 (22,000)	22,000 (500,000)	2-1 composite, Longitudinal	[99]
PZT/Ni_0.7_Zn_0.3_Fe_2_O_4_/Ni	-	20	280	2-2 composite, Transverse	[100]
Terfenol-D/PMN-PT/Terfenol-D	4000	1000	10,300	2-2 composite, Longitudinal	[101]
Terfenol-D/PVDF/Terfenol-D	2300	1000	7930	2-2 composite, Longitudinal, Shear mode	[102]
NCZF/textured 0.675PMN-0.325PT/NCZF	-	1000	1200	2-2 composite, Transverse.	[103]
Ni/Doped-BaTiO_3_	7000	-	7.1	2-2 composite, Longitudinal	[104]
BaTiO_3_/CoFe_2_O_4_ (bulk ceramic)	730	1000	38	Transverse, PLD	[105]
Pb(Zr_0.52_Ti_0.48_)O_3_/CoFe_2_O_4_ (bulk ceramic)	600	1000	155	Transverse, sol–gel	[106]
Pb(Zr,Ti)O_3_/Metglas (bulk foil)	22	1000	7000	Transverse, GSV deposition with laser annealing	[107]
Pb(Zr_0.52_Ti_0.48_)O_3_/Ni (bulk foil)	86	1000	772	Transverse, sol–gel	[108]
BaTiO_3_/Ni (bulk foil)	87	1000	90	Transverse, CSD	[109]

**Table 2:** Room-temperature α_HV_ value of nanostructured magnetoelectric composites.

Nanostructures	*H*_DC_ (Oe)	f_AC_ (Hz)	$\alpha_{V}^{H}(\mathrm{mV}\;{\mathrm{cm}}^{-1}\;{\mathrm{Oe}}^{-1})$	Note	Reference
NCZF/0.8PZT-0.2PZN	2600	1000	150	0-3 composite, Transverse	[110]
0.65Pb(Zr_0.52_Ti_0.48_)O_3_–0.35NiFe_2_O_4_	2500	194	16	0-3 composite, PLD, Longitudinal,	[111]
BiFeO_3_–CoFe_2_O_4_	2900	-	338	0-3 composite, PLD, Transverse	[49]
Pb(Zr_0.52_Ti_0.48_)O_3_–CoFe_2_O_4_	-	1000	220	0-3 composite, sol–gel	[112]
[BaTiO_3_–BiFeO_3_] × 15	-	1000	49,000	2-2 composite, Longitudinal, PLD	[113]
Ir_0.3_Mn_0.7_/FeCoSiB/AlN	-	700	430,000	Transverse, Sputtering	[114]
La_0.7_Sr_0.3_MnO_3_/Pb(Zr_0.52_Ti_0.48_)O_3_	4000	1000	4	Longitudinal, PLD	[115]
NiFe_2_O_4_/BaTiO_3_	100	1000	12	Longitudinal, PLD	[116]
CoFe_2_O_4_/PZT	6000	1000	70	Longitudinal, sol–gel	[117]
CoFe_2_O_4_/BaTiO_3_	100	1000	104	Longitudinal, PLD	[118]
FeCoSiB/AlN	6	100(753)	3100 (737,000)	Transverse, Sputtering	[119]
Ni_0.8_Zn_0.2_Fe_2_O_4_/Pb(Zr_0.6_Ti_0.4_)O_3_	-	1000	15	Longitudinal, PLD	[120]
CoFe_2_O_4_(core)-PZT(shell) nanofiber	2000	-	29,500	Transverse, nanofiber	[121]
PZT-TDE	1500	100	500	1-3 Composite, Longitudinal	[122]
Pb(Fe_0.5_Nb_0.5_)O_3_/Ni_0.65_Zn_0.35_Fe_2_O_4_/Pb(Fe_0.5_Nb_0.5_)O_3_	3000	1000	383	2-2 composite, PLD, Transverse	[65]
PZT-CFO	6000	10–1000	55–5	0-3 composite, PLD, Transverse and Longitudinal	[123]
PZT/NiFe_2_O_4_ (multilayer)	2000	1000	400	2-2 composite, PLD	[124]
